# Adapting environmental surveillance for polio to the need to track antimicrobial resistance

**DOI:** 10.2471/BLT.20.258905

**Published:** 2021-01-07

**Authors:** Christine Årdal, David McAdams, Astrid Louise Wester, Sigrun Møgedal

**Affiliations:** a Antimicrobial Resistance Centre, Norwegian Institute of Public Health, Postboks 222 Skøyen, 0213 Oslo, Norway.; b Fuqua School of Business and Economics Department, Duke University, Durham, United States of America.; c Norwegian Institute of Public Health, Oslo, Norway.

The near eradication of polio, a disease that once paralysed hundreds of thousands of children every year,^1^ is one of humankind’s great successes. Yet the mission to reach all children is incomplete, as wild poliovirus still survives in several districts in Afghanistan and Pakistan. As of 20 October 2020, 132 cases of wild poliovirus have been reported for the year 2020 in these countries, an increase from the 94 cases recorded during the same time period in 2019.^2^ Limitations in vaccine access due to insecurity continue to represent the biggest threat to fully interrupting virus transmission.^3^ The situation is further aggravated by an increase in vaccine-derived poliovirus cases. From 1 January to 5 October 2020, the number of vaccine-derived poliovirus cases (type 2) was 409, already higher than the 378 cases reported for the whole of 2019.^4^

Poliovirus transmission can be stopped if the virus cannot find an unvaccinated person to infect. Yet if children are missed or vaccination routines are interrupted, polio can quickly resurge, making it especially dangerous in conflict-ridden and hard-to-reach areas or in times of pandemic, such as with the current spread of coronavirus disease 2019 (COVID-19). The Global Polio Eradication Initiative temporarily suspended all mass oral polio vaccination campaigns from April through July 2020 due to COVID-19, since these campaigns must be performed door-to-door.^1^ In October 2020, cross-border spread of vaccine-derived poliovirus was increasing, with the potential for an exponential rise in the number of infected children if immunization campaigns do not resume.^4^

The substantial reduction in polio cases over the past several decades has been achieved largely through repeated and systematic oral polio vaccine campaigns, now supplemented with inactivated polio vaccine.^5^ However, eradicating the last poliovirus from the world requires extensive surveillance. The primary polio surveillance method is rapid identification of children having acute flaccid paralysis, collection of their stool samples and subsequent laboratory testing for diagnosis.^6^ Extensive environmental surveillance, that is, testing of sewage samples, is well established and critical to complement acute flaccid paralysis surveillance as well as document the elimination of wild poliovirus. However, the cost of these surveillance systems may be difficult to justify in countries where resources are low and polio is almost non-existent. Multipurposing polio surveillance, that is, integrating other communicable disease surveillance with polio surveillance, could improve sustainability and country engagement.

The *Global polio surveillance action plan, 2018–2020* and the *Polio endgame strategy 2019–2023* call for such long-term integration. This effort should also include expanding the current environmental surveillance network to allow integration with broader communicable disease control efforts and with other public health initiatives such as cholera control and antimicrobial resistance surveillance.

In 2018, the World Health Organization (WHO) and the Global Polio Eradication Initiative identified 29 priority countries, all low- or middle-income countries, where environmental surveillance is needed to ensure that all infections are identified.^6^ In its 2019 status report, the Global Polio Surveillance Action Plan reported that nine countries and one territory in the WHO Eastern Mediterranean Region and 23 countries in the African Region have established environmental surveillance.^6^

Among these 32 countries and one territory performing environmental polio surveillance, 12 do not participate in WHO’s Global Antimicrobial Resistance Surveillance System, meaning that they are likely unaware of current local resistance patterns.^7^ This lack of knowledge can result in suboptimal treatment and unnecessary deaths. Since antimicrobial resistance and poliovirus can be detected through sewage, a significant opportunity to multipurpose environmental surveillance processes exists, thereby strengthening the sustainability of environmental sample collection.

WHO’s Global Antimicrobial Resistance Surveillance System gathers aggregate national laboratory data on antibiotic susceptibility of cultivated isolates from clinical samples. The system focuses on eight bacteria from four clinical sample types and 35 pathogen–antimicrobial combinations of international concern and has been supplemented with a module to report on emerging resistance patterns.^7^ These data show the occurrence of antibiotic resistance in selected populations within health-care institutions, but do not necessarily reflect antimicrobial resistance levels in the broader population. WHO’s Integrated Global Survey on *Escherichia coli* producing extended spectrum β-lactamases (the Tricycle project) is being piloted in six lower-middle-income and low-income countries, and a new module within WHO’s surveillance system is under development for reporting these data. The protocol includes environmental sampling such as from river water near cities.^8^

Countries that do not yet participate in WHO’s antimicrobial surveillance system but do perform environmental polio surveillance could use polio samples for antimicrobial resistance surveillance, to increase data. Additionally, the 21 countries that participate in both the antimicrobial resistance surveillance system and environmental polio surveillance could supplement their data on resistance with population-based data from sewage. Such data can indicate potentially underestimated transmission routes in settings without safe sewage treatment.

Sewage samples can be used to detect pathogen–antimicrobial combinations for five of WHO’s antimicrobial resistance surveillance system bacteria: *Acinetobacter baumannii, Escherichia coli, Klebsiella pneumoniae, Salmonella* spp. *and Shigella* spp. This detection can be done with simple cultivation methods of the same samples used for polio environmental surveillance.

Next-generation sequencing would allow for detection of antimicrobial resistance genes, both as to presence and abundance.^9^ Some researchers estimate that next-generation sequencing can be affordably implemented with all costs for collection, shipment, deoxyribonucleic acid purification, sequencing and bioinformatics analysis of two sewage samples annually from two sites within the same country at 2000 United States dollars.^9^ Moreover, no patient consent is needed for population-based sewage sampling.

Sewage samples can also be expanded to antibiotic residues and to other pathogens of concern. In Pakistan, polio environmental surveillance samples have been tested for severe acute respiratory syndrome coronavirus-2 (SARS-CoV-2).^10^ The WHO Collaborating Centre Risk Assessment of Pathogens in Food and Water is producing a guidance document on harmonizing polio, COVID-19, and antimicrobial resistance wastewater surveillance, which is expected to be piloted in Iraq, Pakistan and South Africa. The detection of antibiotic residues could allow local health authorities to identify areas with the most widespread inappropriate use of second-line and last-resort antibiotics. Educational efforts and other antibiotic-control programmes could then be focused in those areas, and evaluated based on subsequent changes in antibiotic consumption.

The economic incentives of multipurposing environmental surveillance are strong. The most obvious benefit is that surveillance allows health authorities to detect poliovirus, antimicrobial resistance and SARS-CoV-2 at the same time. However, to ensure that low- and middle-income countries have incentive to invest in the surveillance systems needed to monitor such global threats, these systems should also provide actionable intelligence on pathogens of primarily local importance, such as *Vibrio cholerae*, *Giardia* and seasonal influenza.

Additional local benefits arise from being able to monitor the occurrence of resistant bacteria at the local level. Knowing which places have more bacteria resistant to a first-line drug can inform antibiotic prescribing guidelines and decisions as to where to deploy resources, including diagnostics, second-line antibiotics and infection-prevention measures. Such measures include access to vaccines as well as to safe water, sanitation and hygiene services, to reduce the spread of resistant infections and potentially even reverse selection pressure on resistance.^11^

Yet limitations exist. In low-resource settings, every additional financial expenditure risks diverting resources away from essential services, such as surveillance and water, sanitation and hygiene activities.^12^ In addition, many countries have limited sewage facilities. Yet gathering data from even two sites per country would be valuable and provide additional insight into infection prevalence. Technical limitations include the lack of an established quality assurance system for detecting antimicrobial resistance and SARS-CoV-2 in sewage to date, and of standardized protocol for technical handling of the samples and for epidemiological modelling of the results. There are also methodological weaknesses associated with using environmental surveillance for antibiotic consumption surveillance, as well as costly equipment that is not normally present in low-resource settings.

Virtually every country could benefit from increased environmental surveillance, but a logical next step is to multipurpose environmental surveillance in countries already performing polio environmental surveillance, to detect other pathogens in addition to poliovirus. Doing so would increase the sustainability of environmental collection efforts, improving antimicrobial resistance and SARS-CoV-2 surveillance in low-resource settings, and would strengthen efforts to finally eradicate polio.
